# Temporal indexing of medical entity in Chinese clinical notes

**DOI:** 10.1186/s12911-019-0735-x

**Published:** 2019-01-31

**Authors:** Zengjian Liu, Xiaolong Wang, Qingcai Chen, Buzhou Tang, Hua Xu

**Affiliations:** 1 Key Laboratory of Network Oriented Intelligent Computation, Harbin Institute of Technology, Shenzhen, Shenzhen, 518055 China; 2 School of Biomedical Informatics, The University of Texas Health Science Center at Houston, Houston, TX USA

**Keywords:** Temporal indexing, Clinical notes, Recurrent neural network, Convolutional neural network, Medical entity

## Abstract

**Background:**

The goal of temporal indexing is to select an occurred time or time interval for each medical entity in clinical notes, so that all medical entities can be indexed on a united timeline, which could assist the understanding of clinical notes and the further application of medical entities. Some temporal relation shared tasks for the medical entity in English clinical notes have been organized in the past few years, such as the 2012 i2b2 NLP challenge, 2015 and 2016 clinical TempEval challenges. In these tasks, many heuristics rule-based and machine learning-based systems have been developed. In recent years, the deep neural network models have shown great potential on many problems including the relation classification.

**Methods:**

In this paper, we propose a recurrent convolutional neural network (RNN-CNN) model for the temporal indexing task, which consists of four layers: input layer – generates representation for each context word of medical entities or temporal expressions; LSTM (long-short term memory) layer – learns the context information of each word in a sentence and outputs a new word representation sequence; CNN layer – extracts meaningful features from a sentence and outputs a new representation for medical entity or temporal expression; Output layer – takes the representations of medical entity, temporal expression and relation features as input and classifies the temporal relation. Finally, the time or time interval for each medical entity can be directly selected according to the probability of each temporal relation predicted by above model.

**Results:**

To investigate the performance of our RNN-CNN model for the temporal indexing task, several baseline methods were also employed, such as the rule-based, support vector machine (SVM), convolutional neural network (CNN) and recurrent neural network (RNN) methods. Experiments conducted on a manually annotated corpus (including 563 clinical notes with 12,611 medical entities and 4006 temporal expressions) show that RNN-CNN model achieves the best F1-score of 75.97% for temporal relation classification and the best accuracy of 71.96% for temporal indexing.

**Conclusions:**

Neural network methods perform much better than the traditional rule-based and SVM-based method, which can capture more semantic information from the context of medical entities and temporal expressions. Besides, all our methods perform much better for the accurate time indexing than the time interval indexing, so how to improve the performance for time interval indexing will be the main focus in our future work.

## Background

Medical entity, as one of the most important information in the clinical notes, is usually related to the time information. To assist the understanding and application of medical entity, lots of researches were devoted to the temporal relation extraction (including before, after, simultaneous, overlap, etc.) between medical entities and temporal expressions in the clinical notes [1–5]. While the temporal indexing task focuses on medical entity’s temporal relation with temporal expressions, its purpose is to select an accurate occurred time or time interval for each medical entity, i.e. all medical entities can be indexed in the same timeline by linking to different time or time intervals.

In the last few years, several public challenges have been organized for the temporal relation extraction in clinical records. The 2012 i2b2 NLP challenge [5] has set up three subtasks including event detection, temporal expression extraction and temporal relation identification, and also provided a manually annotated corpus with 310 discharge summaries. The clinical TempEval tasks in 2015 [3] and 2016 [2] SemEval challenges also involved the temporal relation identification task with 600 manually annotated clinical notes and pathology reports. Lots of teams from all around the world participated in these challenges, and developed many different kinds of temporal relation identification systems [1, 4, 6–10], like the heuristics rule-based [4] and machine learning-based systems [1, 6, 7, 10]. Many machine learning algorithms have been used in these systems, such as the maximum entropy (MaxEnt), Bayesian, support vector machine (SVM) [11], conditional random fields (CRF) [12], etc.

Recently, the deep neural network (DNN) has been widely used for various kinds of NLP tasks, including the relation classification [13–17], and has shown great potential. In this paper, we also propose a recurrent convolutional neural network (RNN-CNN) method for the temporal indexing task. We treat the temporal indexing task as a selection problem, which contains three steps: 1) generate a set of candidate times for each medical entity by a rule-based method; Then, 2) predict the temporal relation between medical entities and temporal expressions by our RNN-CNN method, which falls in four categories: NONE, SIMULTANEOUS, BEFORE and AFTER; Finally, 3) select the most relevant time or time interval as the final index for each medical entity.

The following sections are organized as: section 2 introduces the method in detail, experiments and results are presented in section 3, section 4 discusses the experimental results and section 5 draws conclusions.

## Methods

**Fig. 1:**
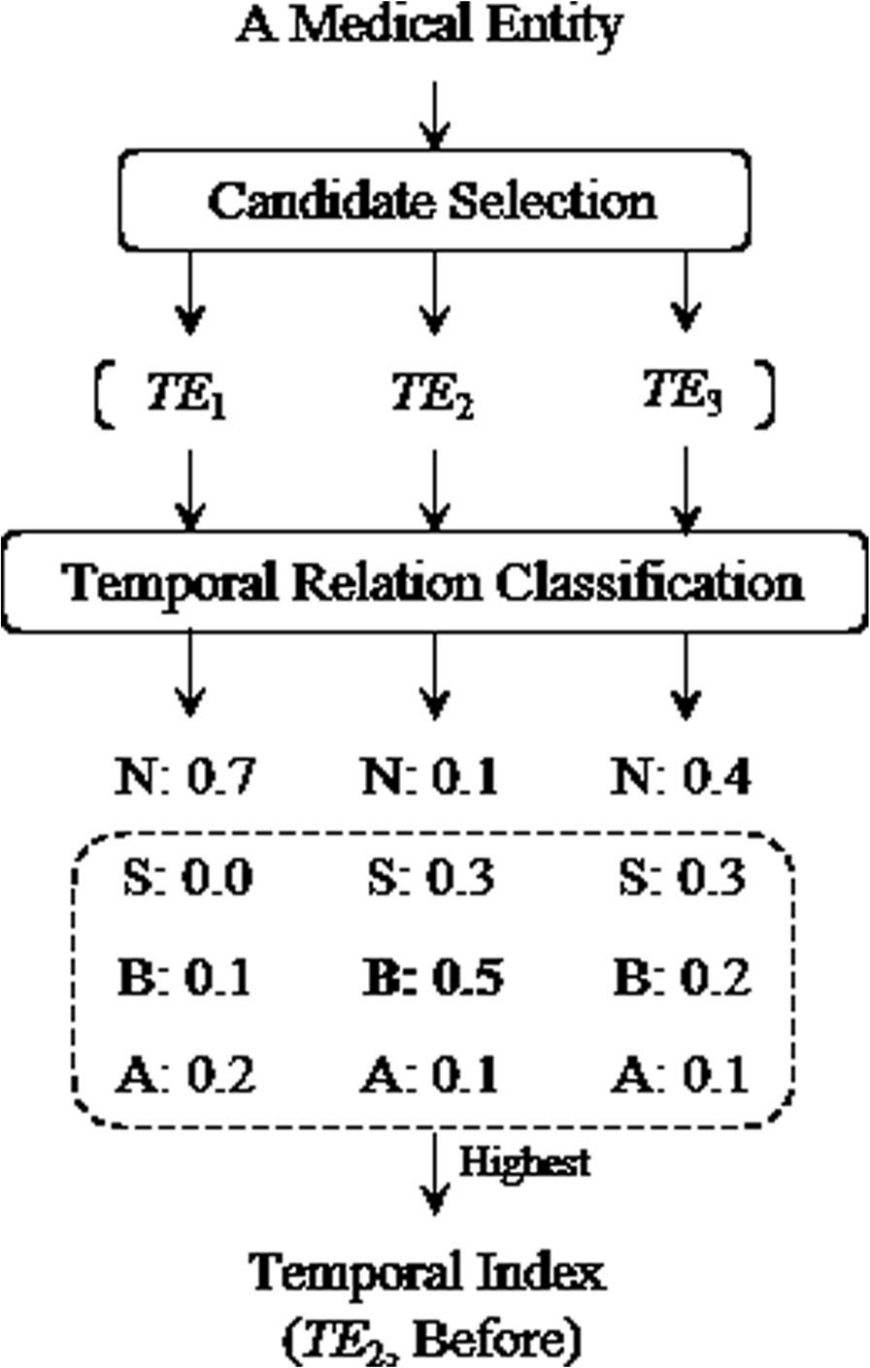
The main flow for temporal indexing

The main purpose of the temporal indexing task is to select a corresponding occurred time or time interval for each medical entity. In this paper, we solve this problem as a pairwise selection problem, which contains three steps: 1) Firstly, generate a set of candidate times for each medical entity, which can reduce number of negative samples, and avoid the imbalance problem of training data. 2) Secondly, predict the temporal relation for each pair of medical entity and temporal expression, which falls in four categories: NONE, SIMULTANEOUS, BEFORE and AFTER, where NONE means there is no relation between the medical entity and temporal expression, SIMULTANEOUS indicates the temporal expression is the indexed time of this medical entity, and BEFORE (AFTER) means the first time interval before (after) the temporal expression is the indexed time interval of this medical entity. 3) Finally, select the most relevant temporal expression from the candidate set by the highest confidence of the relation type: SIMULTANEOUS, BEFORE or AFTER. Figure 1 shows the main flow for temporal indexing, where N, S, B and A represent NONE, SIMULTANEOUS, BEFORE and AFTER resplendently, and the number follow them are their confidences.

### Candidate selection

**Fig. 2:**
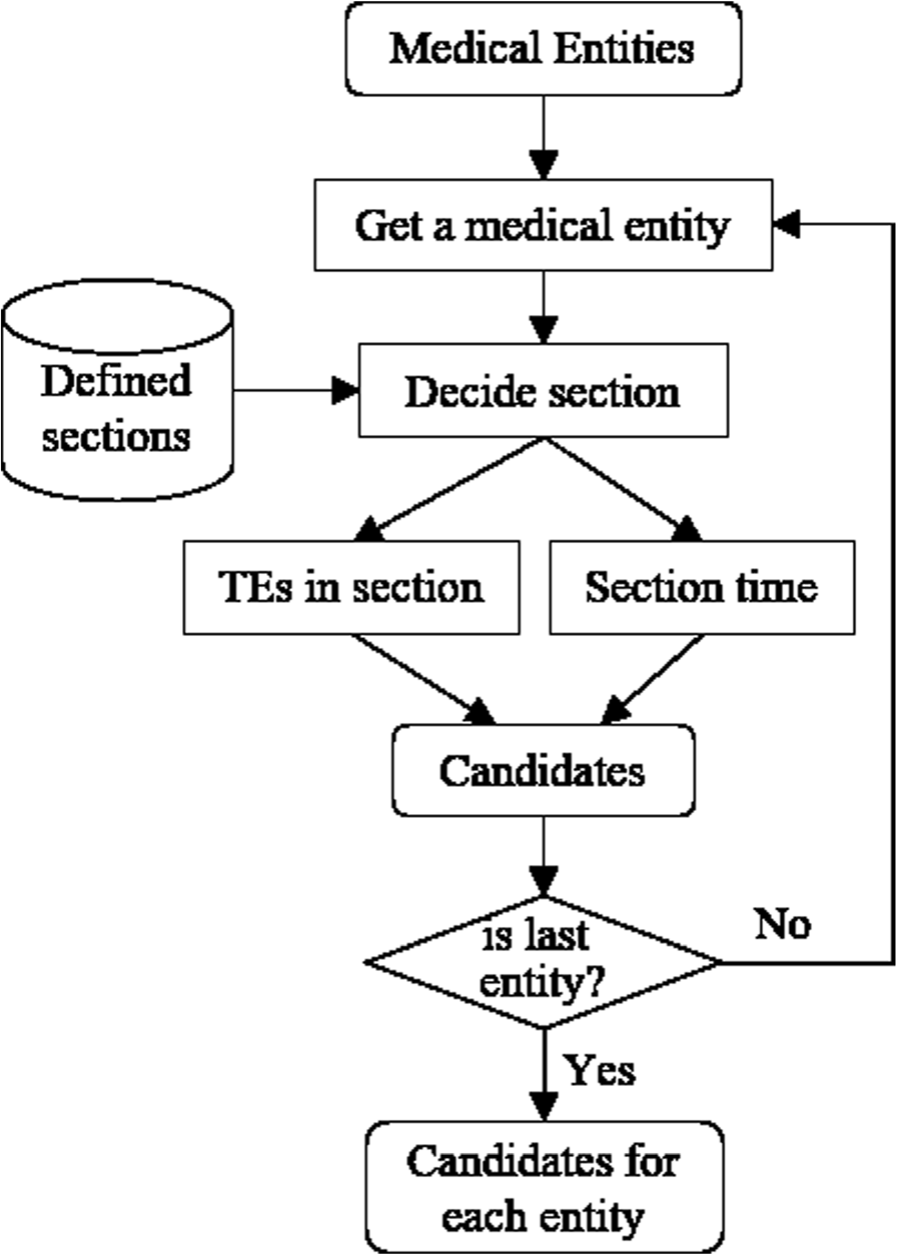
The main flow for candidate selection

As utilizing the pair-wise method to select the temporal index for medical entity, we need pair the medical entity with each temporal expression in the clinical note. However, most of these temporal expressions are not related with current medical entity, which produces many negative samples and causes the data imbalance problem. Therefore, we construct a candidate selection module to generate a much small candidate set for each medical entity. By analyzing the Chinese clinical notes, we find that there are some constant sections in each clinical note, such as the “chief complaint”, “history of present illness”, etc., and these sections are independent of each other. Besides, the occurred times of these sections also are much different, for instance, the medical entities in “history of present illness” section are almost occurred before the admission time, and which in “conditions in discharge” section are occurred at the discharge time. Table 1 lists some sections and their occurred times we summarize in the Chinese clinical notes. Based on this observation, we select the section time and all temporal expressions in corresponding section as the candidate times for each medical entity. Figure 2 shows the main flow for the candidate selection. For each medical entity, we first decide the section it belongs to, then further collect all temporal expressions in this section and corresponding section time (as Table 1) as the final candidates of this medical entity.

**Table 1:** Sections and corresponding occurred times in the Chinese clinical notes

Section name	Occurred time	Relation
Chief complaint, History of present illness, Past medical history, Personal history, Conditions in admission	Admission	Before
Physical examination, Assistant examination, Preliminary diagnosis, Diagnosis on admission	Admission	Simultaneous
Diagnosis and treatment Conditions in discharge, Diagnosis on discharge Discharge orders	Admission Discharge Discharge	After Simultaneous After

**Fig. 3:**
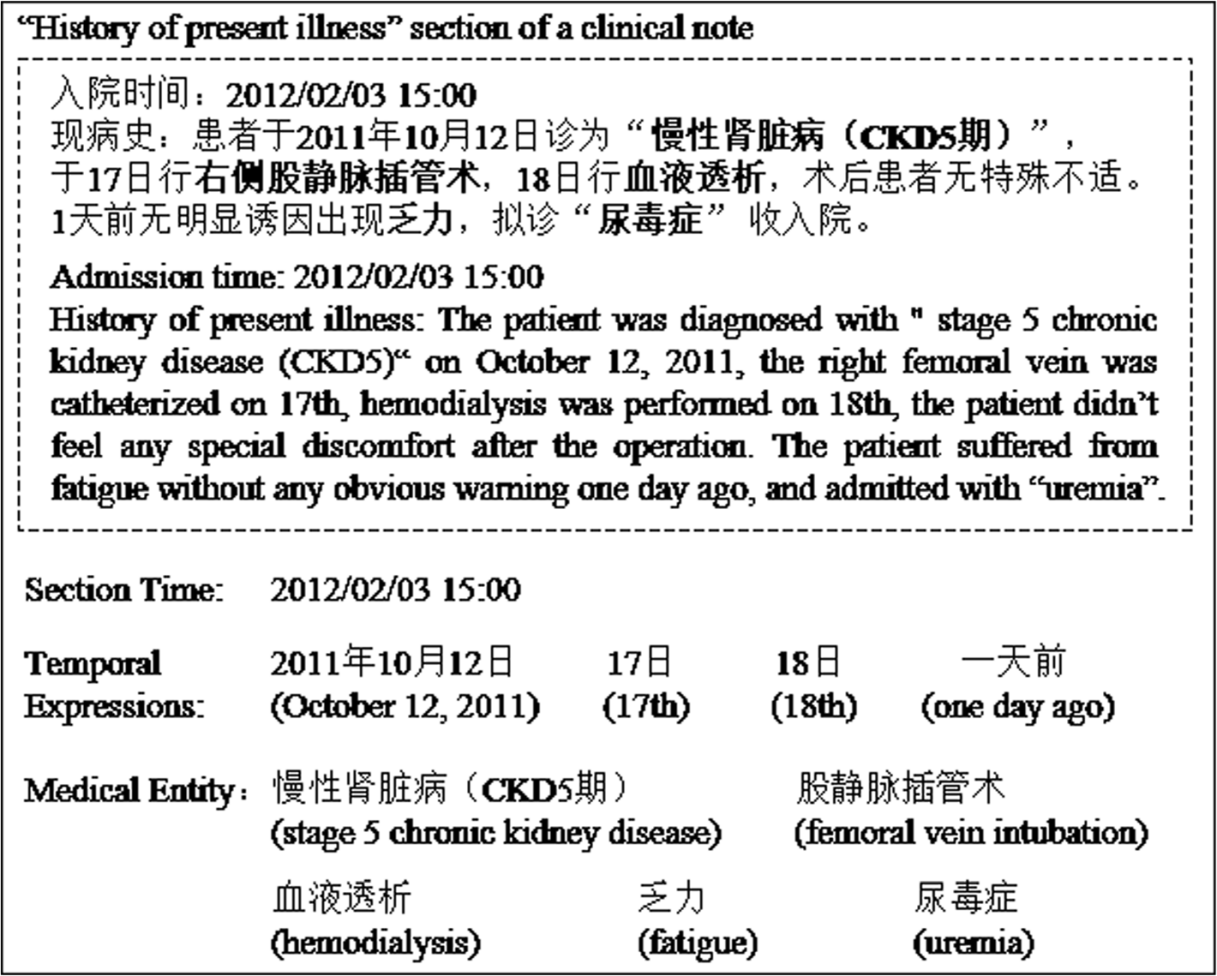
An example for the candidate selection

Figure 3 shows an example for the candidate selection, in which a “History of present illness” section is presented with the section time (also is the admission time), other four temporal expressions, and five medical entities. According to our candidate selection strategy, the section time and four temporal expressions are all selected as the candidates for each medical entity in this section.

### Temporal relation classification

**Fig. 4:**
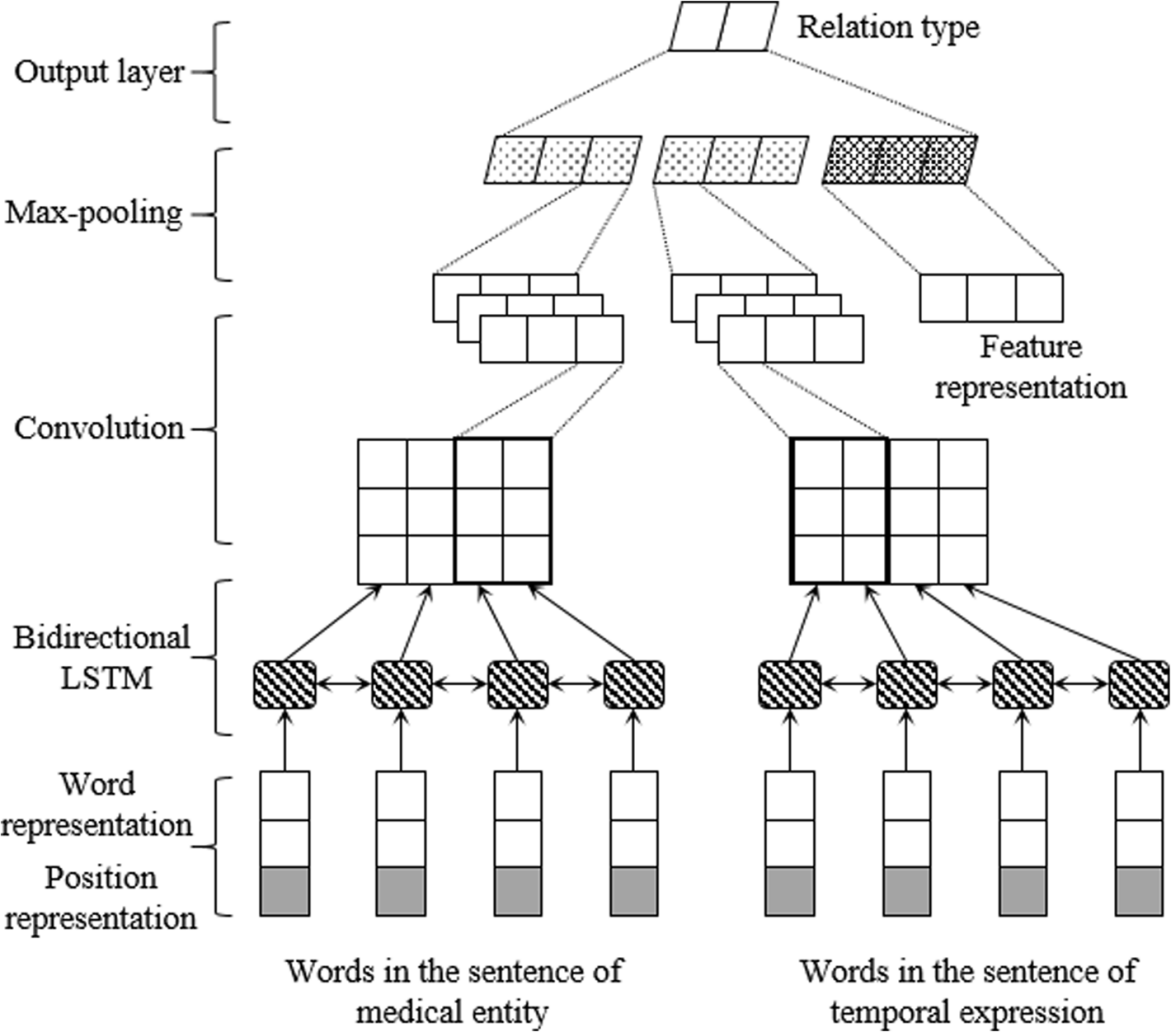
Overview architecture of RNN-CNN

After the candidate selection step, we need further classify the temporal relation (NONE, SIMULTANEOUS, BEFORE and AFTER) of each pair of medical entity and temporal expression, which turn into a relation classification problem. In this paper, a recurrent convolutional neural network (RNN-CNN) model was proposed for this task, as shown in Fig. 4, which contains four main layers: 1) input layer, which takes the sentence of medical entity, the sentence of temporal expression and temporal relation features as input, generates the representation of each word in a sentence and the representation of features. 2) LSTM layer, which includes a forward LSTM and a backward LSTM, takes the word representation sequence of a sentence as input, and outputs a new word representation sequence that captures the context information of each word in this sentence. 3) CNN layer, which takes the word representation sequence of a sentence outputted by LSTM layer as input, generates the representation of medical entity or temporal expression. 4) Output layer, which concatenate the representation of medical entity, temporal expression and relation features together by a hidden layer, and predicts the type of temporal relation by the softmax function.

The main purpose of LSTM and CNN used in our model is to learn a representation for each medical entity and temporal expression from their corresponding sentences. The LSTM is used to learn the context information of each word and the long-distance dependences between words. In other word, the representation of each word output by LSTM not only contains the particular information of current word, but also implies the global information of the sentence. Then, the CNN is applied to extract the significant features from word representation sequence, eliminate the noise and redundant information, and finally generate a representation for each medical entity and temporal expression. More detail introduction of above four layers will present in the following sections.

### Input layer

Our RNN-CNN model learns the representations of medical entity and temporal expression from the sentences where they belong to respectively. Give a sentence *S* = (*w*_1_, *w*_2_, ⋯, *w*_*n*_) with each word *w*_*t*_ (1 ≤ *t* ≤ *n*), which contains the medical entity (or temporal expression) word *w*_*k*_, *P* = (*d*_1_, *d*_2_, ⋯, *d*_*n*_) is the sequence of positions for each word, where *d*_*t*_ = *t* − *k* (1 ≤ *t* ≤ *n*). Then, the representation *x*_*t*_ of word *w*_*t*_ can be calculated by:

1 $$ {x}_t=\left[{E}_w\bullet \overrightarrow{w_t},{E}_d\bullet \overrightarrow{d_t}\right] $$

Where *E*_*w*_ and *E*_*d*_ are the embedding matrixes for words and positions, $\overrightarrow{w_t}$ and $\overrightarrow{d_t}$ are the one-hot vectors for word *w*_*t*_ and position *d*_*t*_ respectively. The *E*_*w*_ matrix is initialized by a pre-trained word embedding learned from a large-scale of unlabeled data by the word2vec tool [18], while the *E*_*d*_ matrix is randomly initialized from a uniform distribution ranging in [− 1, 1]. We can see that, *x*_*t*_ is generated by concatenating the word representation and position representation, which can capture the semantic information and distance information of the words respectively. The final word representation sequence *X* = (*x*_1_, *x*_2_, ⋯, *x*_*n*_) is fed to the next LSTM layer. Same way is used to initialize the medical entity sentence and temporal expression sentence respectively.

Except the context words of medical entity and temporal expression, our RNN-CNN model also employed lots of temporal relation features. Give a feature set *G*, *g*_*i*_ ∈ *G* (1 ≤ *i* ≤ *m*) is a feature from it, *m* is the number of features. Then, the representation of feature *g*_*i*_ is:

2 $$ {z}_i={E}_g\bullet \overrightarrow{g_i} $$

where *E*_*g*_ is the embedding matrix for relation features, which is randomly initialized from a uniform distribution ranging in [− 1, 1], $\overrightarrow{g_i}$ is the one-hot vector for feature *g*_*i*_. The final feature representation is *Z* = (*z*_1_, *z*_2_, ⋯, *z*_*n*_), which concatenates all the representation of each feature together, and is utilized to predict the temporal relation in output layer. The main temporal relation features used in this paper include:

- The section name of medical entity.
- The section name of temporal expression.
- Whether the medical entity and temporal expression are in the same section.
- Whether the temporal expression is admission time, discharge time or others.
- Whether there is any temporal expression in the sentence of medical entity.
- The number of temporal expression in the sentence of medical entity, is 0, 1 or more.
- Whether the medical entity and temporal expression are in the same sentence.
- The temporal expression is front or behind the medical entity if they are in the same sentence.
- Whether the temporal expression sentence is front or behind the medical entity sentence.
- In medical entity section or sentence, where there is any temporal expression front the medical entity.

### LSTM layer

The LSTM layer takes a word representation sequence *X* = (*x*_1_, *x*_2_, ⋯, *x*_*n*_) as input and produces a new representation sequence *H* = (*h*_1_, *h*_2_, ⋯, *h*_*n*_), where *h*_*t*_ = [*h*_*ft*_, *h*_*bt*_] (1 ≤ *t* ≤ *n*) is a concatenation of the outputs of both forward LSTM *h*_*ft*_ and backward LSTM *h*_*bt*_ at step *t*. More specifically, an LSTM unit is composed of an input gate *i*_*t*_, a forget gate *f*_*t*_, an output gate *o*_*t*_ and a memory cell *c*_*t*_, which takes *x*_*t*_, *h*_*t* − 1_ and *c*_*t* − 1_ as input at step *t* and produces *h*_*t*_ and *c*_*t*_ as following formulas:

$$ {i}_t=\upsigma \left({W}_{xi}{x}_t+{W}_{hi}{h}_{t-1}+{W}_{ci}{c}_{t-1}+{b}_i\right) $$

$$ {f}_t=\upsigma \left({W}_{xf}{x}_t+{W}_{hf}{h}_{t-1}+{W}_{cf}{c}_{t-1}+{b}_f\right) $$

3 $$ {c}_t={f}_t\otimes {c}_{t-1}+{i}_t\bigotimes \mathit{\tanh}\left({W}_{xc}{x}_t+{W}_{hc}{h}_{t-1}+{b}_c\right) $$

$$ {o}_t=\upsigma \left({W}_{xo}{x}_t+{W}_{ho}{h}_{t-1}+{W}_{co}{c}_t+{b}_o\right) $$

$$ {h}_t={o}_t\bigotimes \mathit{\tanh}\left({c}_t\right) $$

Where σ is the element-wise sigmoid function, ⨂ is the element-wise product, *W*_*i*_, *W*_*f*_, *W*_*c*_ and *W*_*o*_ (with subscripts: *x*, *h* and *c*) are the weight matrices for input *x*_*t*_, hidden state *h*_*t*_ and memory cell *c*_*t*_ respectively, *b*_*i*_, *b*_*f*_, *b*_*c*_ and *b*_*o*_ denote the bias vectors.

### CNN layer

Convolutional neural network (CNN) [19] is able to extract primary information in a sentence, which takes the word representation sequence *H* = (*h*_1_, *h*_2_, ⋯, *h*_*n*_) outputted by LSTM layer as input and produces a new representation for this sentence. We suppose that there are *L* convolution filters (feature maps) *W*^(*i*)^ (1 ≤ i ≤ *L*), in which the filter size is *k* × *d*, *k* is the context window size of the filter, *d* is the dimension of word representation *h*_*t*_. For each filter *W*^(*i*)^, we can get *n* − *k* + 1 features (*n* is the length of sentence) by multiplying with each *k*-length sliding window in *H*, as following formula:

4 $$ {C}_j^{(i)}=\sigma \left({W}^{(i)}\bigotimes {H}_{\left[j:j+k-1\right]}+{b}^{(i)}\right)\ \left(1\le j\le n-k+1\right) $$

Where ${C}_j^{(i)}$ is the *j*-th feature extracted by filter *W*^(*i*)^ from the context matrix [*h*_*j*_, *h*_*j* + 1_, ⋯, *h*_*j* + *k* − 1_], σ is element-wise sigmoid function, ⨂ is the element-wise product, *b*^(*i*)^ is the bias vector for *i*-th filter. Then, all the features extracted by filter *W*^(*i*)^ from the word representation sequence *H* are ${C}^{(i)}=\left({C}_1^{(i)},{C}_2^{(i)},\cdots, {C}_{n-k+1}^{(i)}\right)$.

After the convolution operation, we further apply a max-pooling operation to pick out the most significant feature from all the features extracted by filter *W*^(*i*)^, which is denoted as:

5 $$ {C}_{max}^{(i)}=\mathit{\max}\left\{{C}_1^{(i)},{C}_2^{(i)},\cdots, {C}_{n-k+1}^{(i)}\right\} $$

After all *L* convolution filters are applied for the feature extraction, we can get the final sentence representation $C=\left[{C}_{max}^{(1)},{C}_{max}^{(2)},\cdots, {C}_{max}^{(L)}\right]$.

### Output layer

By applying above LSTM and CNN layers to the sentences, we can get two sentence representations *C*_*M*_ and *C*_*T*_ for medical entity and temporal expression respectively. Then, the output layer employs a fully-connected network with softmax activation function to concatenate the medical entity representation *C*_*M*_, temporal expression representation *C*_*T*_ and relation feature representation *Z* as input, and outputs the probabilities of each relation class as follow:

6 $$ Y= softmax\left({W}_o\bullet \left[{C}_M,{C}_T,Z\right]+{b}_o\right) $$

Where *y*_*i*_ ∈ *Y* (1 ≤ *i*≤| *Y*| ) is the probability for relation class *i*, ∣*Y*∣ is the number of relation class, *W*_*o*_ is the weight matrix in output layer, and *b*_*o*_ is the bias vector.

## Results

### Dataset and annotation

**Fig. 5:**
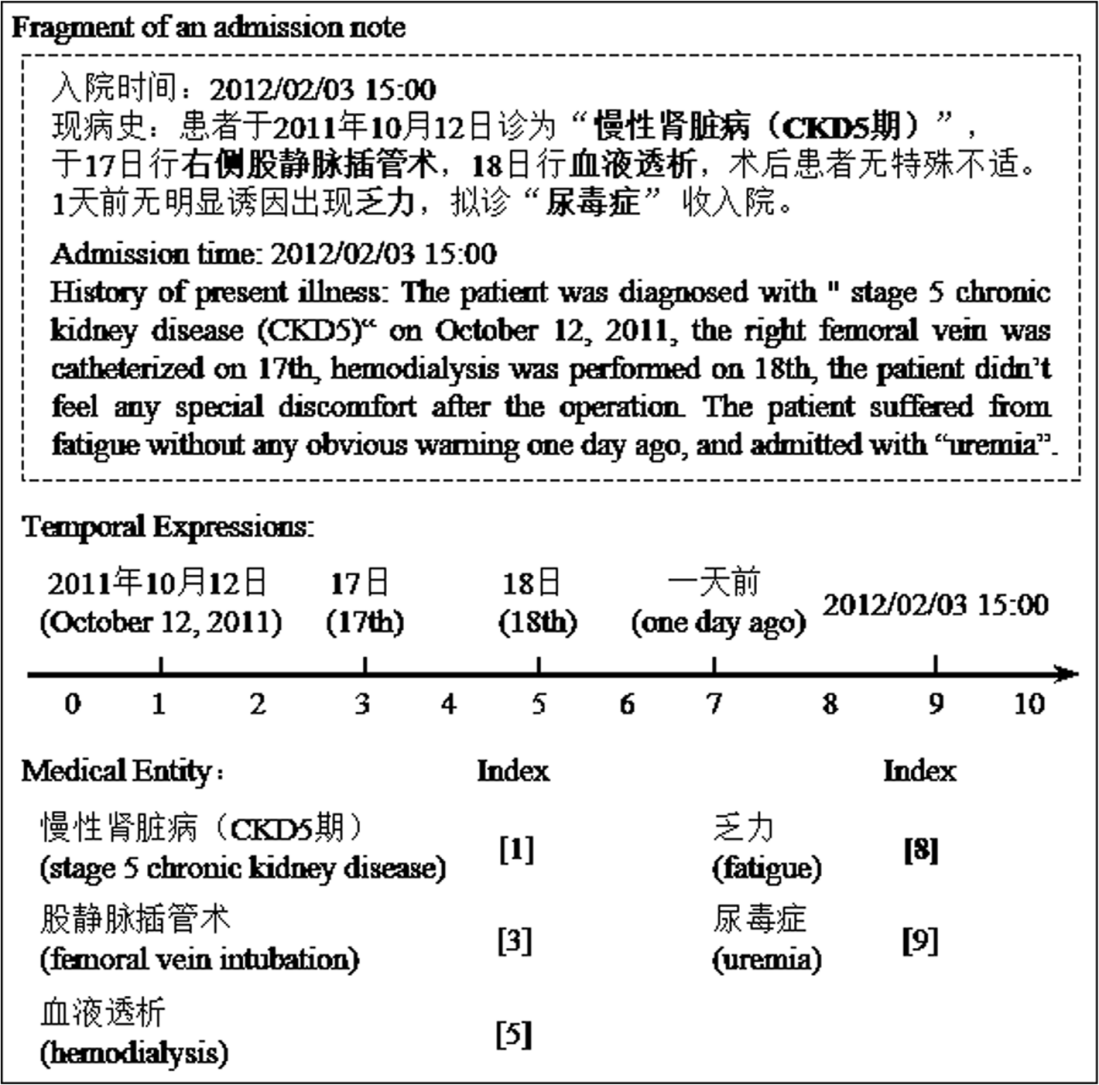
Examples for the annotation of temporal indexing

A real world dataset of 563 de-identified clinical notes collected from a tier A hospital of China was first manually annotated with medical entities (ME) and temporal expressions (TE). Then, four master students with computer science and medical informatics background were recruited to annotate the temporal index for each medical entity in the clinical notes by following steps: 1) All TEs in a clinical note were sorted by their normalization value. 2) Then, all the times (TEs) and time intervals (between two adjacent TEs) were allocated with a unique ID number orderly. For example, the time interval before the first TE was numbered to ‘0’, while the first TE was ‘1’. 3) Finally, the annotators should select a corresponding occurred time or time interval for each medical entity, and annotate them a unique ID number as the temporal index. Some examples were shown in Fig. 5.

In Fig. 5, we can see that there is a phrase of an admission note which states the admission time (February 3rd, 2012) of a patient at first sentence, and describes patient’s medical history at next paragraph. As the medical history, the patient was diagnosed with “stage 5 chronic kidney disease (CKD5)” at last year (October 12, 2011), and further received some treatments (like right femoral vein and hemodialysis) at next few days. Then a new followed sentence further described the patient suffered from fatigue one day ago, and admitted as “uremia” again. To analyze the semantic of those sentences, the baseline time of “one day ago” is no longer same with last sentence (last year - 2011), which actually means the day before the admission time (February 3rd, 2012). Therefore, the corresponding time of “uremia” is the admission time, not the last year. This example shows the importance of implicit semantic information for the temporal indexing task.

To calculate the inter-rater annotation agreement of these four annotators, we ask them to annotate 100 out of 563 notes at the same time. Actually, each annotator only need to annotate 50 notes, that each notes only be annotated twice. When there is any disagreement between these four annotators, a new judge was brought in to select or confirm the different annotations. Based on these 100 notes, inter-rater annotation agreements of the temporal indexing are 0.7130 by using Kappa statistics, and 0.7425 by using accuracy, which indicates that our annotation was reliable. Besides, the annotation of temporal indexing is much difficult since the corresponding time of medical entity need annotators to inference from the context, and the semantic ambiguity would cause many errors of annotation.

After annotation, we obtain a medical entity temporal indexing dataset with 563 records, 12,611 medical entities, 4006 temporal expressions, and corresponding temporal indexes for each medical entity. Among these temporal indexes, 8592 medical entities were annotated to time nodes, and 4019 medical entities were annotated to time intervals. The annotated dataset was randomly divided into training and test sets. The training set consisted of 413 records with 9348 medical entities and 2982 temporal expressions, while the test set consisted of 150 records with 3263 medical entities and 1024 temporal expressions. The statistics of the dataset were shown in Table 2 in detail, where “#*” denotes the number of “*”.

**Table 2:** Statistics of the annotated dataset for temporal indexing of medical entity

	#Record	#ME	#TE	#Time node	#Time interval
Training set	413	9348	2982	6461	2887
Test set	150	3263	1024	2131	1132
Total	563	12,611	4006	8592	4019

### Experimental setup and evaluation

#### Baseline

In this paper, we also employ several baseline methods for the temporal indexing task as follow:

- Rule-based method (Rule), which bases on the section head listed in Table 1, and takes some rules like: Select the section time as the temporal index if there is no any temporal expression in current section; Select the nearest front temporal expression as the index in current sentence, etc.

- Support vector machine-based method (SVM), which takes the context words of medical entity, context words of temporal expression, and relation features as input, and trained by the support vector machine algorithm.

- Recurrent neural network-based method (RNN), which is much similar to the RNN-CNN model, concatenated the representations of last two words in the forward and backward LSTM as the sentence representation instead of using the CNN layer.

- Convolutional neural network-based method (CNN), which is also similar to the RNN-CNN model except it did not employ the LSTM layer to learning the context information and directly put the word representation sequence into the CNN layer.

#### Metrics

To evaluate the performance of our methods, we firstly use the Precision (P), Recall (R) and F1-score (F1) criteria on the temporal relation classification task, in which a relation pair is correctly predicted only when its relation type is same with the gold type. Then, we further use accuracy to evaluate the performance of our methods on the finial temporal indexing task under two criteria: “Relaxed”, which indicates that the temporal index of a medical entity is correct only when the temporal expression selected is same with the gold one; “Strict”, which means that the temporal index is correct only when the temporal expression selected is same with the gold one and also with the same type (i.e. the predicted time or time interval for medical entity is correct). The “Strict” criterion is the primary one.

#### Hyper parameters

In this paper, we propose a recurrent convolutional neural network to classify the temporal relation of medical entity and temporal expression, which use the stochastic gradient descent (SGD) algorithm and cross entropy loss function for the parameter estimation with hyper-parameters as shown in Table 3, which refers to our previous experiences and other relevant works [13–17]. For the fairness of comparison between different methods, all the neural network-based used the same hyper-parameters in Table 3.

**Table 3:** Hyper-parameters used for the neural network

Hyper-parameter	Value
Dimension of word representation	50
Dimension of position representation	30
Dimension of feature representation	20
Size of LSTM unit	50
Size of convolution filter	3/5/7
Number of convolution filters	100
Max length of the sentence	100
Dropout probability	0.5
Batch size	32
Training epochs	20

### Experimental results

Table 4 shows the results of our rule-based candidate time selection method for medical entity. We can see that the candidate time sets selected by our method have cover 96.22 and 97.18% medical entities on the training set and test set respectively. Besides, there are total 31,156 and 10,233 candidates on training set and test set, and about 3 candidates for each medical entity averagely. These indicate that our candidate selection method can cover the correct candidate time for most of the medical entity with a much smaller candidate set, which obviously reduces the number of negative samples and avoids the data imbalance problem.

**Table 4:** The results of our candidate selection method

	Selected TEs	Selected MEs	Coverage Scale
Training set	31,156	8995	96.22%
Test set	10,233	3171	97.18%

The micro-average precisions, recalls and F1-scores of various methods for the temporal relation classification task are listed in Table 5, where “Merged” denotes that all the predictions of four machine learning-based methods (SVM, CNN, RNN and RNN-CNN) are merged together by calculating the average probability for each relation type. Among the top three individual methods, the two neural network-based methods (CNN and RNN) achieve much higher F1-scores of 74.86 and 74.87% than the SVM-based method of 71.63%. Further comparing the CNN and RNN methods, CNN has a much higher precision of 70.68%, while RNN has a much higher recall of 84.68%. By jointing the RNN and CNN neural network together, the F1-score of our RNN-CNN method is further improved to 75.97%, which outperforms all above individual methods by over 1.10%. Finally, after merging the predictions of all above methods, the F1-score for the temporal relation classification is improved to 78.36% much higher than the RNN-CNN method by 2.39%, and the precision also is obviously improved by over 3.56%. These further indicate that the “Merged” method can take the full advantages of each above method.

**Table 5:** The performance of various methods for the temporal relation classification (%)

Method	Precision	Recall	F1-score
SVM	70.13	73.18	71.63
CNN	70.68	79.56	74.86
RNN	67.10	84.68	74.87
RNN-CNN	71.41	81.15	75.97
Merged	74.97	82.07	78.36

Table 6 lists the accuracies of various methods for the temporal indexing task under both “Relaxed” and “Strict” criteria. We can see that the rule method achieves a better accuracy than SVM under the “Relaxed” criterion, but its accuracy under “Strict” criterion is much lower than SVM, which indicates that the rule method has a poor ability to classify the temporal relation. Among the four machine learning-based methods, RNN-CNN method achieves the best accuracy of 88.26 and 71.10% for the temporal indexing under “Relaxed” and “Strict” criterion respectively, which demonstrates that RNN-CNN method has the better performance for the temporal relation classification after jointing the CNN network to the RNN method. Finally, the accuracies under both “Relaxed” and “Strict” criteria are further improved by merging the results of all above machine learning-based methods (“Merged”), which reach to 88.57 and 73.31% respectively.

**Table 6:** The accuracies of various methods for the temporal indexing (%)

Method	Relaxed	Strict
Rule	86.42	67.36
SVM	85.53	69.32
CNN	86.33	69.84
RNN	87.13	71.38
RNN-CNN	88.26	71.96
Merged	88.57	73.31

## Discussion

To further analyze the gold test set, we find that there are total 3263 medical entities, 2131 out of them were indexed to the time node (relation type is SIMULTANEOUS), and the remaining 1132 medical entities were indexed to the time interval (relation type is BEFORE or AFTER). In order to investigate the performances of our various methods on these two kinds of temporal indexing, we list the precisions, recalls and F1-scores of them in Table 7. We can see that the precisions of rule and SVM methods for the time node indexing and the recalls of them for time interval indexing are much lower than other methods, which indicates that more medical entities were wrongly indexed to the time node by rule and SVM methods. This also is the reason why the SVM method achieves the highest recall for time node indexing but also has the lowest precision. Among the three neural network-based methods, RNN achieves much higher F1-scores than CNN for both time node and time interval indexing, and RNN-CNN further outperforms the RNN methods by F1-scores of 0.12 and 0.85% respectively. Besides, RNN-CNN also achieves a best precision of 74.42% for the time node indexing, and best recall of 58.22% for the time interval indexing. Finally, the “Merged” method still achieves the best F1-scores of 76.66 and 63.61% for two kinds of temporal indexing respectively among all our methods. The significant contribution made by “Merged” method is the obvious improvement of F1-score for time interval indexing by 2.68%. For all methods, the F1-scores of them for the time node indexing are much higher than the time interval indexing, and the difference between them is minimized by the “Merged” method.

**Table 7:** The performances of various methods on the time and time interval indexing (%)

Method	Time node (%)			Time interval (%)		
	Precision	Recall	F1-score	Precision	Recall	F1-score
Rule	69.62	79.35	74.17	60.79	44.79	51.58
SVM	65.79	83.39	73.55	86.30	42.84	57.26
CNN	72.02	79.35	75.50	64.26	51.94	57.45
RNN	74.13	78.41	76.21	65.21	58.13	61.47
RNN-CNN	74.80	78.84	76.76	65.68	59.01	62.17
Merged	73.38	81.23	77.10	73.12	58.39	64.93

Analyzing the temporal indexing results of each method, there are two type of errors: first is the selection error, which means the method selected a wrong temporal expression for current medical entity. Another one is the type error, which means the method has selected a correct temporal expression for current medical entity but with a wrong temporal relation type. Firstly, comparing the SVM-based method with our RNN-CNN method, we find that there are 515 selection errors and 495 type errors caused by SVM respectively, 383 and 532 caused by RNN-CNN. The number of selection errors of SVM is much larger than RNN-CNN by 132, which is the main reason why the RNN-CNN method has a better performance of temporal indexing. For example, in sentence “患者于2011-9-13诊断为右输尿管上段结石,于2011-9-15行右输尿管镜钬激光碎石术。” (“The patient was diagnosed as a right ureteral calculus on September 13, 2011, and the right ureteroscopy holmium laser lithotripsy was performed on September 15, 2011.”), two medical entities “right ureteral calculi” and “right ureteroscopy holmium laser lithotripsy” were all indexed to temporal expression “September 13, 2011” by the SVM-based method, while the RNN-CNN correctly indexed them to “September 13, 2011” and “September 15, 2011” respectively.

On the other hand, the numbers of selection error and type error caused by rule-based method are 443 and 622 respectively. We can see that the number of type error of rule-based method is much larger than the RNN-CNN method by 90, which indicates that rule-based method has much worse performance for the classification of temporal relation. In other word, large number of rules and patterns can select a correct temporal expression for most of medical entities, but extract the implicit semantic information and classify the type of temporal relation is much difficult for them. For example, in sentence “18日行刮宫术,术后予以防感染治疗。” (“The uterine curettage was performed at 18th, and the anti-infection treatment was given after the operation.”), the treatment “anti-infection” was given after the 18th, so the temporal relation between them is “AFTER”, but was predicted as “SIMULTANEOUS” by the rule-based method.

Similarly, the selection errors of RNN and CNN methods are 420 and 446 respectively, both larger than the RNN-CNN method by 37 and 63 respectively, which indicates that the combined utilization of both RNN and CNN could learn more implicit semantic information than each of them, and improve the performance of temporal relation classification and temporal indexing for each medical entity.

Finally, all the results of four machine learning-based methods are merged together by calculating the average probability of each relation type. We can see that the “Merged” method performed a much better performance of temporal indexing than all other single methods, and also has much less selection and type errors than RNN-CNN method by 10 and 34 respectively. This indicates that merging the results of all single methods can further improve the performance of temporal indexing, especially for the classification of temporal relation.

In addition, we also employ many hand-crafted semantic features in our RNN-CNN model. To investigate the effects of these features, we further evaluate the pure RNN-CNN model without any manual features on the test set, and the performance of it is 53.78 and 68.46% under “Strict” and “Relaxed” criteria respectively, which is much lower than the RNN-CNN with manual features. Therefore, we can see that the hand-crafted semantic features still play a critical role for the temporal indexing of medical entity.

Although our RNN-CNN method has presented a much valuable performance for the temporal indexing of medical entity, there are also some complex situations that our method did not handle very well, especially for the estimation of global semantic relation. For example, a phrase of history of present illness section in admission note “患者2011年10月9日出现发热,伴右侧腰痛。4天前症状加重并伴有恶心、呕吐。遂来我院就诊,以急性肾盂肾炎收入院。” (“The patient appeared fever with right lumbago on October 9, 2011. The symptoms were aggravated with nausea and emesis at 4 days ago. Hence, the patient was admitted with acute pyelonephritis.”), our method can correctly index the “fever”, “lumbago” to “October 9, 2011”, and “nausea”, “emesis” to “4 days ago”, but wrongly index the “acute pyelonephritis” to “4 days ago”. In fact, the occurred time of disease “acute pyelonephritis” was the admission time appeared at the head of admission note. To estimate this temporal relation needs more global semantic information that was the main direction in our future work.

## Conclusions

In this paper, the temporal indexing task was treated as a selection problem: compare the medical entity with each candidate time firstly, then select the most relevant time or time interval as the final index for this medical entity. We first construct a temporal indexing corpus manually, which contains 563 Chinese clinical notes with 12,611 medical entities and 4006 temporal expressions. Then, a recurrent convolutional neural network (RNN-CNN) method is proposed for the temporal indexing, which achieves the best F1-score of 75.97% for temporal relation classification, and best accuracy of 71.96% for temporal indexing on the independent test set when comparing with some baseline methods. Finally, the performance of temporal indexing task is further improved by merging the predictions of all machine learning-based methods. However, we also find that our methods perform much worse for the time interval indexing, which will be the main focus in our future work.

## Abbreviations

CNN: Convolutional neural network
CRF: Conditional random fields
RNN: Recurrent neural network
RNN-CNN: Recurrent convolutional neural network
SGD: Stochastic gradient descent
SVM: Support vector machine
